# Urgent care center wait times increase for COVID-19 results in August 2020, with rapid testing availability limited

**DOI:** 10.1186/s12913-021-06338-y

**Published:** 2021-04-08

**Authors:** Laurie C Yousman, Akshay Khunte, Walter Hsiang, Siddharth Jain, Howard Forman, Daniel Wiznia

**Affiliations:** 1 Yale University School of Medicine, New Haven, USA; 2 Yale University School of Medicine, Department of Radiology, New Haven, USA; 3 Yale University School of Medicine, Department of Orthopaedics and Rehabilitation, 800 Howard Avenue, New Haven, Connecticut 06520 USA

**Keywords:** COVID-19, COVID-19 testing, Urgent care center, Access to care

## Abstract

**Background:**

In a response to the pandemic, urgent care centers (UCCs) have gained a critical role as a common location for COVID-19 testing. We sought to characterize the changes in testing accessibility at UCCs between March and August 2020 on the basis of testing availability (including rapid antigen testing), wait time for test results, cost of visits, and cost of tests.

**Methods:**

Data were collected using a secret shopper methodology. Researchers contacted 250 UCCs in 10 states. Investigators used a standardized script to survey centers on their COVID-19 testing availability and policies. UCCs were initially contacted in March and re-called in August. T-tests and chi-square tests were conducted to identify differences between March and August data and differences by center classification.

**Results:**

Our results indicate that both polymerase chain reaction (PCR) tests to detect COVID-19 genetic material and rapid antigen COVID-19 tests have increased in availability. However, wait times for PCR test results have significantly increased to an average of 5.79 days. Additionally, a high proportion of UCCs continue to charge for tests and visits and no significant decrease was found in the proportion of UCCs that charge for COVID-19 testing from March to August. Further, no state reported a majority of UCCs with rapid testing available, indicating an overall lack of rapid testing.

**Conclusions:**

From March to August, COVID-19 testing availability gradually improved. However, many barriers lie in access to COVID-19 testing, including testing costs, visit costs, and overall lack of availability of rapid testing in the majority of UCCs. Despite the passage of the CARES Act, these results suggest that there is room for additional policy to improve accessibility to testing, specifically rapid testing.

## Introduction

With the onset of the COVID-19 pandemic, urgent care centers (UCCs) have become a common access point for testing,[1, 2] and over the last few months the availability and cost of COVID-19 testing at UCCs have evolved.[3, 4] In particular, the Coronavirus Aid, Relief, and Economic Security (CARES) Act was passed in March 2020 with the goal of expanding access to testing by providing coverage of COVID-19 testing for all patients, regardless of insurance status.

Existing literature has explored many aspects of the COVID-19 testing landscape, including addressing rapidly increasing demand for tests, lack of testing resources, supply chain barriers, and long turnaround times.[5, 6] Several different locations for COVID-19testing have also been highlighted by the existing literature, including physician offices, in-patient hospital settings, emergency rooms, community health centers, walk-in retail clinics, and urgent care centers.[7] Additionally, the cost of COVID-19 testing has been reported for top US hospitals, and has further been broken down into average cost by different testing providers.[7, 8].

While existing research delves into the barriers that impact lack of accessibility to COVID-19 testing,[9] there are many gaps in the literature, including studies with a direct focus on UCCs. To our knowledge, no such study has examined COVID-19 testing at urgent care centers and specifically compared availability of testing during time periods before and after the passage of the CARES Act. Additionally, while some literature points out the extra barriers that uninsured patients may face when seeking testing and care for COVID-19,[10, 11] there is a lack of existing literature specifically focused characterizing the multiple cost barriers to testing for uninsured patients in light of the evolving costs of COVID-19 testing. We examined how availability of testing, wait times for test results, and cost of testing have changed at UCCs from March 2020 to August 2020 in the United States.

## Methods

This study received IRB exemption from the Yale School of Medicine. We utilized a secret shopper methodology outlined in previous studies.[12–14] UCCs were defined as walk-in clinics separate from hospital-affiliated or freestanding emergency departments. Due to many states exhibiting extremely caseloads,[15] researchers focused on states considered COVID-19 hotspots. 25 UCCs were randomly selected from each of the 10 states with the highest COVID-19 caseloads in March 2020[16] using the Solv Health Urgent Care Directory,[17] containing approximately 11,000 UCCs across the United States. In each individual state, every UCC in the directory was assigned a numeric value and a random number generator was used to select 25 UCCs from each state.

Investigators called UCCs posing as uninsured patients seeking COVID-19 testing. A standardized script was used to inquire about availability of testing, estimated test result wait time, and whether testing and UCC visits were free for uninsured patients. Rapid testing was defined as a < 2 h wait time. As the rapid antigen test is often conducted in conjunction with the PCR test and is less reliable than the PCR test,[18] it was measured as a separate variable and not included in the August calculation of estimated wait time. The same 250 UCCs were contacted initially in March 2020 and called again in August 2020.

Each UCC was classified into one of four center types: (1) Non-affiliated, defined as a stand-alone or chain of UCCs with no affiliation to a private practice or hospital network, (2) Extension of Private Practice, defined as a UCC that is operated by and connected to a private practice, (3) Extension of Hospital/Health Network, defined as a UCC associated with a non-academic hospital or health network, or (4) Academic, defined as a UCC associated with a teaching hospital. The primary outcome variable was the availability of COVID-19 testing at centers in March and August. Additionally, data were collected on center requirements for testing, center policy on the cost of testing before and after the passage of the CARES Act, and expected wait time for test results.

Statistical analysis was performed using JMP Pro Version 15. Comparisons between March and August calls and comparisons between testing availability by center type were conducted using 2 sample t-tests and chi-square tests to generate differences and odds ratios (OR), respectively. For odds ratios, the non-exposure group was considered March while the exposed group was considered to be August. The outcomes were each of the first four variables listed in Table 1. *P*-values lower than 0.05 were considered statistically significant, therefore, 95 % confidence intervals (CI) were used.

**Table 1:** Variables Characterizing Accessibility of COVID-19 Testing at UCCs

	Proportion % (n)	Univariate odds ratio (95 % CI)	*P* value
**Testing Available**			
March (*n* = 250)	24.00 % (60)	Ref	
August (*n* = 250)	66.00 % (165)	4.43 (1.99, 9.84)	0.0001
**If Testing Available, Only Testing Symptomatic Patients**			
March (*n* = 60)	98.33 % (59)	Ref	
August (*n* = 163)	22.70 % (37)	0.0050 (0.0007, 0.0372)	< 0.0001
**If Testing Available, Charge for Test**			
March (*n* = 53)	86.79 % (46)	Ref	
August (*n* = 153)	83.66 % (128)	0.78 (0.32, 1.92)	0.5874
**If Testing Available, Charge for Visit**			
March (*n* = 59)	94.92 % (56)	Ref	0.0008
August (*n* = 157)	74.52 % (117)	0.16 (0.05, 0.53)	
	**Mean (days)**	**Difference (95 % CI)**	***P*value**
**If Testing Available, Result Wait Time**			
March (*n* = 52)	4.87	Ref	0.0169
August (*n* = 136)	5.79	+ 0.92 (0.17, 1.67)	

Comparisons between March and August data were conducted. There were 10 states originally contacted in March and re-called in August.

## Results

Table 1 lists changes in testing accessibility from March to August. There was a significant increase in availability of general PCR testing (OR 4.43, 95 % CI 1.99–9.84, p-value = 0.0001). Rapid testing was unavailable in March. In August, some rapid testing was available (8.40 % (*n* = 21)). There was no significant change in the proportion of UCCs charging for testing (OR 0.78, 95 % CI 0.32, 1.92, *p*-value = 0.5874). There was a decrease in the proportion charging for visits (OR 0.16, 95 % CI 0.05, 0.53, *p*-value = 0.0008). The proportions of centers charging for both visits and tests remained high in August. Wait times for non-rapid test results significantly increased from March to August (0.92 days, 95 % CI 0.17–1.67, *p*-value = 0.0169) to an average of 5.79 days.

Table 2 lists changes in testing accessibility from March to August for each individual state surveyed. State level results reveal that testing availability significantly increased in all 10 states. There was an extremely low prevalence of rapid testing available, with no state indicating a majority of centers offering rapid testing and four out of ten states having no centers that offered rapid testing. Wait times for PCR test results did not decrease significantly in any individual state, and increased significantly in Florida.

**Table 2:** State-Level UCC COVID-19 Testing Availability and Wait Times

	Testing Available			Rapid Testing Available (August)	Mean Wait Time (days)		
**State (*n* = 25)**	**March**	**August**	***P*-Value**		**March**	**August**	***P*-Value**
California	4 % (1)	28 % (7)	0.0206	0 % (0)	N/A	6.81	-
Colorado	8 % (2)	48 % (12)	0.0016	4 % (1)	4.50	5.00	0.6158
Florida	32 % (8)	64 % (16)	0.0235	8 % (2)	4.00	7.12	0.0304
Georgia	8 % (2)	68 % (17)	< 0.0001	16 % (4)	5.00	5.29	0.5762
Illinois	24 % (6)	72 % (18)	0.0007	16 % (4)	4.92	4.33	0.1873
Louisiana	16 % (4)	76 % (19)	< 0.0001	28 % (7)	5.75	4.32	0.1263
Massachusetts	40 % (10)	92 % (23)	< 0.0001	12 % (3)	5.80	6.20	0.8790
New Jersey	32 % (8)	60 % (15)	0.0470	0 % (0)	5.19	7.53	0.1448
New York	40 % (10)	76 % (19)	0.0099	0 % (0)	4.80	6.24	0.4738
Washington	36 % (9)	76 % (19)	0.0044	0 % (0)	3.79	4.75	0.3669

State level of characterizations of testing availability and mean wait time were conducted for the 10 states contacted in March and August. Rapid testing was not available in March and was only characterized in August

Table 3 displays differences in PCR testing availability by center classification. In a comparison between all four center types, there was a significant difference. Non-affiliated UCCs demonstrated the highest rates of PCR testing availability, while extension of private practice UCCs demonstrated the lowest.

**Table 3:** Testing Availability by Center Classification

	Proportion of Centers Offering Testing % (n)	Odds Ratio (95 % CI)	*P*-value
**PCR Testing Availability**			0.0077
Non-affiliated (*n* = 143)	70.63 % (101)	Ref	
Extension of Private Practice (*n* = 38)	42.11 % (16)	3.31 (1.58, 6.91)	0.0015
Extension of Hospital/Health Network (*n* = 59)	71.19 % (42)	0.973 (0.50, 1.90)	0.9369
Academic Hospital (*n* = 10)	60.00 % (6)	1.60 (0.43, 5.97)	0.4819

Individual urgent care centers were classified into one of four categorizations. Chi-square analysis was only performed on availability of PCR testing and not rapid testing due to low prevalence of rapid testing in each center type

## Discussion

While accessibility of COVID-19 testing at UCCs has slowly improved since March, over a third of UCCs still do not offer testing. Additionally, there is still a severe lack of rapid testing, with only 8.40 % of centers able to provide rapid testing in August. Additionally, wait times for test results have significantly increased between March and August, indicating an insufficiency in the capacity to process tests in the United States.

The CARES Act was intended to increase accessibility of COVID-19 testing,[19] however, our findings indicate that the CARES Act has not decreased the cost of COVID-19 testing for uninsured patients at UCCs. The large majority of UCCs continue to charge for both testing and visits, contradictory to the aims of the CARES Act, and this may discourage patients from pursuing testing. In particular, this continued charge for COVID-19 testing may disproportionately affect uninsured and underinsured individuals.

The prevalence of uninsured individuals and those who cannot afford testing may be compounded by the weakened economy, hampered by the pandemic. In 2019, 9.2 % of people were uninsured, and 55.4 % of people had employer-provided health coverage.[20] As record numbers of Americans lose their jobs,[21] the already significant proportion of the population that is uninsured or in poverty will continue to climb, exacerbating cost barriers to COVID-19 testing. Uninsured status has already been proven to be a health risk for a multitude of healthcare disparities,[22] and it seems likely that this issue will be compounded on several fronts by the COVID-19 pandemic and continued cost barriers to testing.

Limitations include our focus on UCCs in specific states, which may not fully represent testing capabilities across the entire United States. Additionally, not all states have gone through waves of the pandemic concurrently, so the March versus August comparison may compare testing accessibility with varying levels of regional demand.

In conclusion, as greater than five-day old test results have limited clinical utility, future research should examine policy and legislation that can advances the deployment of rapid testing.

## Data Availability

The datasets used and/or analyzed during the current study are available from the corresponding author on reasonable request.

## Abbreviations

UCC: Urgent care center
